# Utilizing Frémy's Salt to Increase the Mechanical Rigidity of Supramolecular Peptide-Based Gel Networks

**DOI:** 10.3389/fbioe.2020.594258

**Published:** 2021-01-05

**Authors:** Galit Fichman, Joel P. Schneider

**Affiliations:** Chemical Biology Laboratory, National Cancer Institute, National Institutes of Health, Frederick, MD, United States

**Keywords:** hydrogel, peptide, self-assembly, Frémy's salt, crosslinking, quinone

## Abstract

Peptide-based supramolecular gels are an important class of biomaterials that can be used for biomedical applications ranging from drug delivery to tissue engineering. Methodology that allows one to readily modulate the mechanical properties of these gels will allow yet even a broader range of applications. Frémy's salt is an inorganic salt and long-lived free radical that is known to oxidize phenols. Herein, we show that Frémy's salt can be used to dramatically increase the mechanical rigidity of hydrogels formed by tyrosine-containing self-assembling β-hairpin peptides. When Frémy's salt is added to pre-formed gels, it converts tyrosine residues to o-quinones that can subsequently react with amines present within the lysine side chains of the assembled peptide. This results in the installation of chemical crosslinks that reinforce the gel matrix. We characterized the unoxidized and oxidized gel systems using UV-Vis, transmission electron microscopy and rheological measurements and show that Frémy's salt increases the gel rigidity by nearly one order of magnitude, while retaining the gel's shear-thin/recovery behavior. Thus, Frémy's salt represents an on-demand method to modulate the mechanical rigidity of peptide-based self-assembled gels.

## Introduction

**Figure 1:**
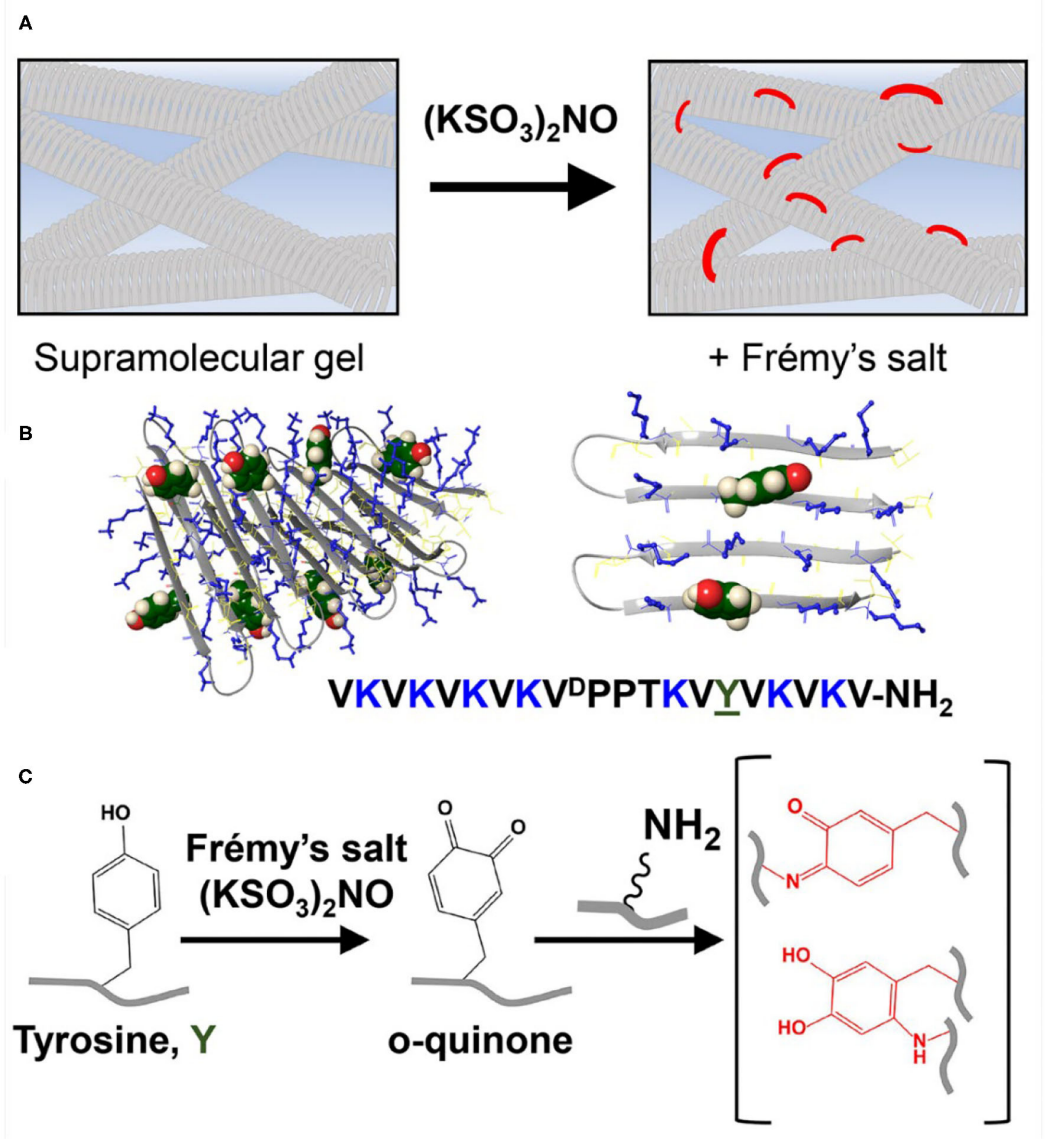
**(A)** Conceptual scheme showing reinforcement of a supramolecular gel scaffold by introducing covalent crosslinks into a non-covalent network using Frémy's salt. **(B)** A tyrosine-functionalized lysine-rich peptide gelator. This peptide self-assembles into a bi-layered cross β-sheet fibrillar network, adopting an amphiphilic β-hairpin conformation in its self-assembled state. At left, eight peptides are shown as part of a fibril. At right, only two adjacent peptides from one monolayer are shown so that the relative positions of the tyrosine and lysine residues can be seen more clearly. Tyrosine side chains are displayed from the hairpin's hydrophilic face and thus the solvent-exposed surfaces of the fibrils. Tyrosine residues are shown in green CPK rendering and lysine side chains are shown as blue sticks. **(C)** The addition of Frémy's salt to the pre-formed gel results in chemical conversion of tyrosine to ortho-quinone, enabling the subsequent formation of covalent crosslinks within the network via the reaction of lysine and ortho-quinone functional groups.

Hydrogels are considered promising biomaterials for various biomedical and biotechnological applications (Kopecek and Yang, 2007). Peptide-based supramolecular hydrogels formed by self-assembly (Fichman and Gazit, 2014; Du et al., 2015; Draper and Adams, 2017; Raymond and Nilsson, 2018; Li et al., 2019) represent one class that has been proven to be suitable for applications such as drug delivery (Li and Mooney, 2016; Li et al., 2016; Majumder et al., 2018), wound healing (Carrejo et al., 2018; Zhou et al., 2019; Thota et al., 2020), and tissue engineering, serving as extracellular matrix mimetics for cell growth and differentiation (Kisiday et al., 2002; Jayawarna et al., 2009; Collier et al., 2010; Alakpa et al., 2016; Ghosh et al., 2017; Hellmund and Koksch, 2019). In specific applications, the ability to modulate and fine tune the mechanical properties of these gels is highly desirable. For example, Alakpa et al. have shown that tuning the hydrogel stiffness can direct perivascular stem cell differentiation, where the expression of neural, chondrogenic, or osteogenic markers were observed according to gel rigidity (1, 13, and 32 kPa, respectively) (Alakpa et al., 2016). Indeed, the ability to modify the mechanical properties of peptide-gels has become an impetus in peptide molecular design (Pashuck et al., 2010; Geisler and Schneider, 2012; Micklitsch et al., 2015; Clarke et al., 2018; Basavalingappa et al., 2019; Hiew et al., 2019). Moreover, efforts have been made to improve the mechanical properties of established supramolecular peptide-based gels, focusing mainly in developing methods to enhance the mechanical rigidity of the gel, as these gels are typically only moderately stiff (Yan and Pochan, 2010; Li et al., 2014). Such efforts include, among others, the introduction of crosslinks into the gel network (Hu et al., 2019) using physical (Greenfield et al., 2010; DiMaio et al., 2017; Bairagi et al., 2019; Scelsi et al., 2019), enzymatic (Bakota et al., 2011; Li et al., 2013), or chemical (Seow and Hauser, 2013; Khalily et al., 2015) crosslinking mechanisms. Interestingly, only limited success has been reported for chemical crosslinking of peptide-based gels (Li et al., 2014). However, one example was recently described by Wang and co-workers, who used a photocrosslinking approach to improve the mechanical stability of tyrosine-containing gels, based on the ruthenium complex (Ru(bpy)_3_Cl_2_)-catalyzed conversion of tyrosine to di-tyrosine that occurs upon light irradiation (Ding et al., 2013). Since tyrosine-containing peptide materials are widely used in various applications (Lee et al., 2019), we wanted to further expand the arsenal of chemical crosslinking strategies that exploit the intrinsic chemistry of phenolic tyrosine. Here, we present a simple *in vitro* post-self-assembly approach to introduce covalent crosslinks into the gel network using potassium nitrosodisulfonate (Frémy's salt), Figure 1A, a long-lived free radical that has been used to chemically convert tyrosine to reactive o-quinone for many decades (Zimmer et al., 1971). The use of Frémy's salt to oxidize tyrosine residues complements the use of enzymes (e.g., tyrosinase). However, Frémy's salt may provide an advantage for use on an established gel network. In contrast to enzymes whose diffusion could be limited if a gel's mesh size is small, nitrosodisulfonate can better penetrate the network.

We had previously developed a class of amphiphilic peptides that can undergo triggered self-assembly into supramolecular hydrogels (Schneider et al., 2002). These peptide gelators are well-characterized over several length scales. For example, using cryo-TEM, Pochan et al. showed that soon after self-assembly is triggered, clusters of well-defined fibrils are formed throughout the sample volume (Yucel et al., 2008). Individual clusters contain dangling fibril ends that grow and interpenetrate neighboring clusters as the network evolves. The exact time at which the clustered fibril network percolates the entire sample volume and the solution becomes a gel is fast (<1 min at 1 wt% peptide) and concentration dependent (Veerman et al., 2006). After the gel point, the network continues to grow, filling the voids, to further rigidify the gel. Cryo-TEM suggests that the final network contains fibrils that entangle and form branch-points, both are physical crosslinks that help define the gel's mechanical properties. The mesh size of the network can be varied (~20–50 nm) by adjusting the peptide concentration or the rate of self-assembly (Ozbas et al., 2004; Branco et al., 2009). TEM and AFM were used to characterize the local morphology of the fibrils affording width and height measurements of 3 and 2 nm, respectively, suggesting that the peptide folds and is stacked when assembled. Solid state NMR later showed that these amphiphilic peptides do indeed fold into a well-defined β-hairpin conformation in their self-assembled state, forming a bilayer cross-β structure within each fibril (Nagy-Smith et al., 2015). Importantly, the fibrils formed are monomorphic indicating that the peptide used in the NMR study, and presumably others in this hairpin class, assembles into a unique arrangement within the fibril. Thus, a structure-based approach can be used to design new gelators that display regiospecific functionality within the fibrils they form, ultimately imbibing targeted properties to the gel. Here, a new peptide gelator is designed to display both lysine and tyrosine residues on the solvent-exposed surface of the fibrils it ultimately forms (Figure 1B). As will be shown, on demand addition of Frémy's salt to the newly-designed gel system results in a chemical conversion of the phenolic-tyrosine into a highly reactive o-quinone (Zimmer et al., 1971). In the lysine rich environment of the assembled peptide, o-quinone can readily react with amines present within the lysine side chains, introducing covalent crosslinks to the system (Yang et al., 2014), Figure 1C. These covalent crosslinks directly contribute to the cohesion of the network, increasing the mechanical rigidity of the gel.

## Results and Discussion

**Figure 2:**
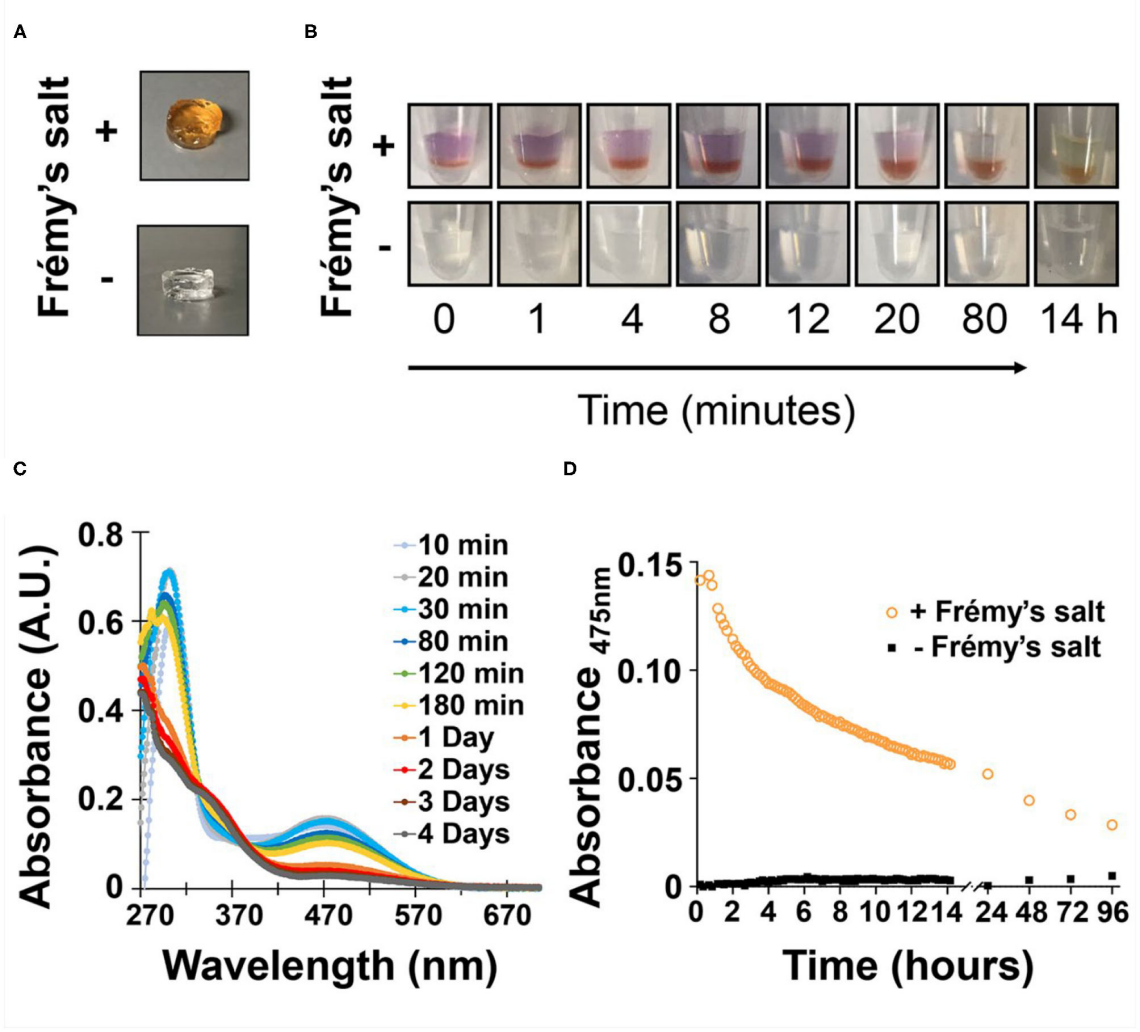
**(A)** Images of pre-formed gels following overnight incubation in the presence or absence of Frémy's salt. Changes in gel color observed only in the presence of Frémy's salt overtime, were monitored by **(B)** the naked eye as well as **(C)** UV-Vis spectroscopy. **(D)** Change in gel absorbance at 475 nm as a function of time and presence or absence of oxidant.

The designed peptide incorporates nine valine residues that drive self-assembly via the hydrophobic effect, arranged in an amphiphilic (AB)_n_ repeat with lysine, where A is a hydrophobic residue and B is hydrophilic with the exception of a sole tyrosine residue at position 15. Based on the previously determined solid-state NMR structure (Nagy-Smith et al., 2015), the peptide should self-assemble as depicted in the model shown in Figure 1B. The model at left shows only a portion of a fibril containing eight folded peptides where each peptide adopts an amphiphilic β-hairpin conformation. Peptides are assembled into a bilayer, sequestering their valine-rich faces from water. The hydrophilic face of each peptide displays its lysine and tyrosine side-chains into solvent and are thus accessible to chemical reagents. The panel at right at Figure 1B shows only two peptides within the fibril to better visualize the relative position of tyrosine and lysine residues. This 20-residue peptide is readily dissolved in water, where repulsive interactions between the protonated lysine side chains prevent it from self-assembling. Peptide assembly is triggered by the addition of saline buffer (pH 7.4), which increases the solution pH and ionic strength, reducing the inter-lysine electrostatic interactions. Assembly leads to a bi-layered cross β-sheet fibrillar network, giving rise to a macroscopic self-supporting gel. Again, since both tyrosine and lysine side chains are displayed from the hairpin's hydrophilic face, those residues are repetitively presented along the solvent-exposed surfaces of the fibrils within the gel system. Having the functional residues exposed to the solvent within the gel matrix provides one the ability to increase gel rigidity on demand, by simply adding Frémy's salt exogenously to a pre-formed gel. We first examined macroscopically how the addition of the oxidant affects the pre-formed gel (Figure 2A). We observed that the addition of the salt did not affect the gel's integrity, as it retained its macroscopic 3D shape. However, when Frémy's salt was added to the colorless gel, the material eventually turned an orange-like color. The observed color change represents a clear indication of tyrosine oxidation. Figure 2B shows the time-dependent coloration of gel, supporting the assertion that an oxidative process within the gel is occurring. When Frémy's salt is initially dissolved in buffer, it is purple in color due to the presence of monomeric nitrosodisulfonate ions. Overtime, the nitrosodisulfonate ions decompose and the purple color of the Frémy's salt solution is discharged (Murib and Ritter, 1952), resulting in a light yellowish solution (Supplementary Figures 1, 2). In the first 20 min following the addition of Frémy's salt solution, the purple color of the solution on top of the gels is clearly observed. Interestingly, during this time period we also observed an initial red layer at the gel-solution interface, at the upper part of the gel. Frémy's salt is a radical that is known to selectively oxidize phenol to quinones (Zimmer et al., 1971). The initial conversion of phenol to o-quinone is typically characterized with the appearance of a red color that later dissipates, giving rise to other secondary colored products (Siegel and Siegel, 1958). The rapid red coloration suggests that the tyrosine residues found at the gel's surface are immediately oxidized by the Frémy's salt solution. As time passes, the oxidant penetrates the gel, completely permeating the material in 20 min, with the entire gel turning red. The change in gel color after an overnight incubation from a red to an orange-yellowish color indicates that the quinone further reacted, forming other end products. UV-Vis spectroscopy was also used to follow the oxidation reaction (Figure 2C). In these studies, to avoid light scattering, a lower peptide concentration of 0.125 wt% was used. At this concentration, the peptide assembles into fibers but does not form gel. In the absence of Frémy's salt, the UV-Vis spectra of the tyrosine-functionalized peptide in HEPES buffer is characterized by a single peak with λ_max_ around 278 nm whose wavelength maximum does not change over 4 days (Supplementary Figure 3). In contrast, we observe that in the presence of the Frémy's salt solution, initial stages of the reaction are characterized by the formation of dopachrome intermediate products, showing absorption maxima at 300 and 475 nm (Dukler et al., 1971). This spectrum resembles other spectra reported in the literature that describe the oxidation of NH_2_-terminal tyrosine and tyrosine-containing peptides either by Frémy's salt (Dukler et al., 1971) or enzymatically by tyrosinase (Yasunobu et al., 1959). After about 30 min both the 300 and 475 nm peaks start to gradually decrease (Figure 2D, Supplementary Figure 4A), and a shoulder around 350 nm appears (Figure 2C, Supplementary Figure 4B). Similar spectral changes were reported previously where Frémy's salt was used to oxidize oligopeptides that contain both lysine and tyrosine residues in their sequence (Wilchek and Miron, 2015). After 1 day the spectrum ceased to change and remained constant for at least 3 days, the last time point assessed. From the UV-Vis data and the qualitative color-change observations we conclude that the oxidation reaction and subsequent crosslinking reactions are complete following an overnight incubation. We envision that the majority of covalent crosslinks are introduced to the system by the reaction of o-quinone with lysine, given its large concentration in the network. It is possible that dityrosine adducts could also form. However, within a given fibril, tyrosine residues are not close enough to react, Figure 1B (right panel) so any dityrosines formed would do so from the reaction of residues located on different fibrils that have entangled within the network.

**Figure 3:**
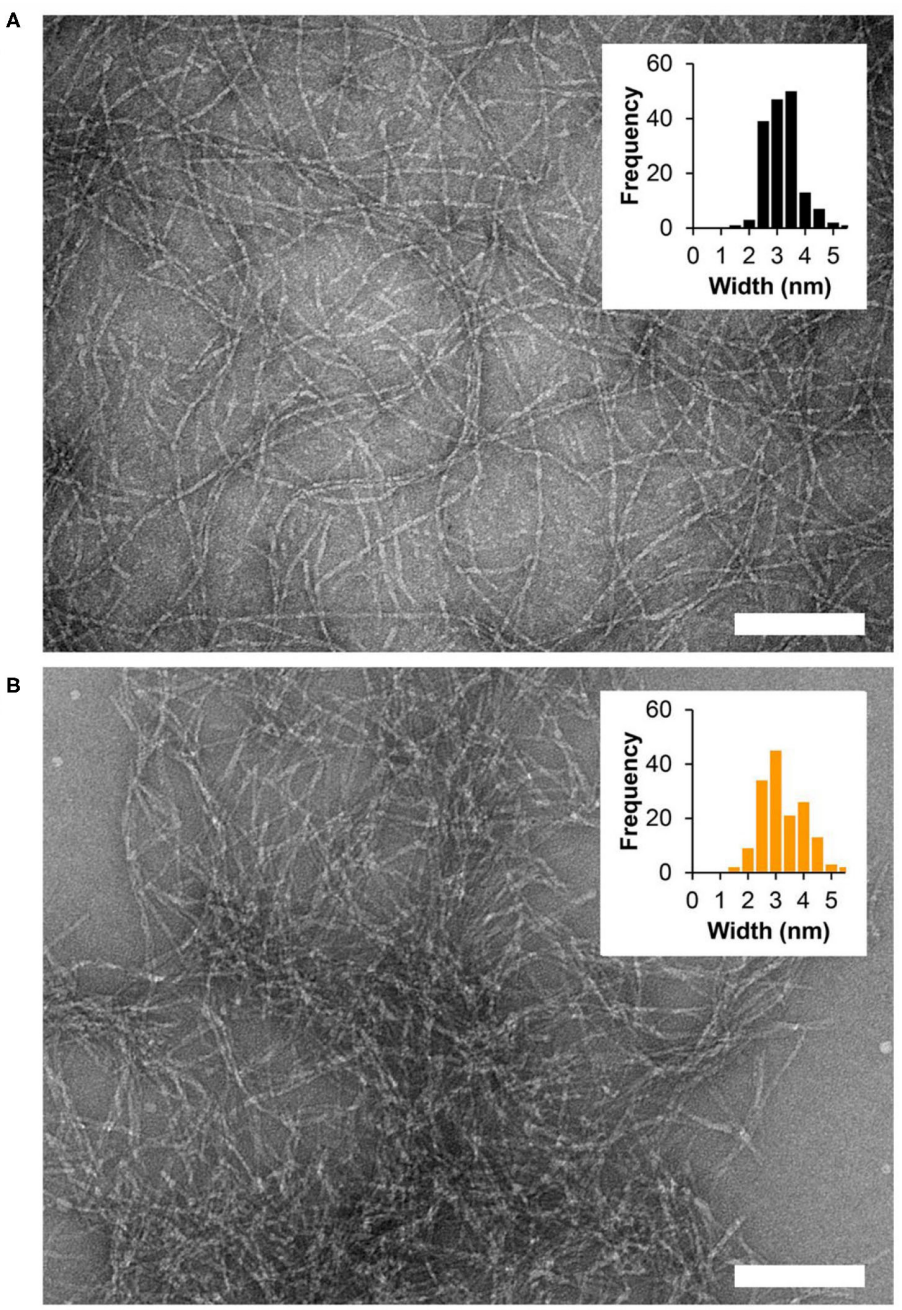
TEM micrographs showing fibrils isolated from 1 wt% fibrillar gel networks **(A)** in the absence of oxidant or **(B)** following overnight oxidation with Frémy's salt. Scale bar = 100 nm. Widths of individual fibrils were determined using ImageJ software, *n* = 164 and *n* = 156 for the non-oxidized and oxidized gel, respectively.

**Figure 4:**
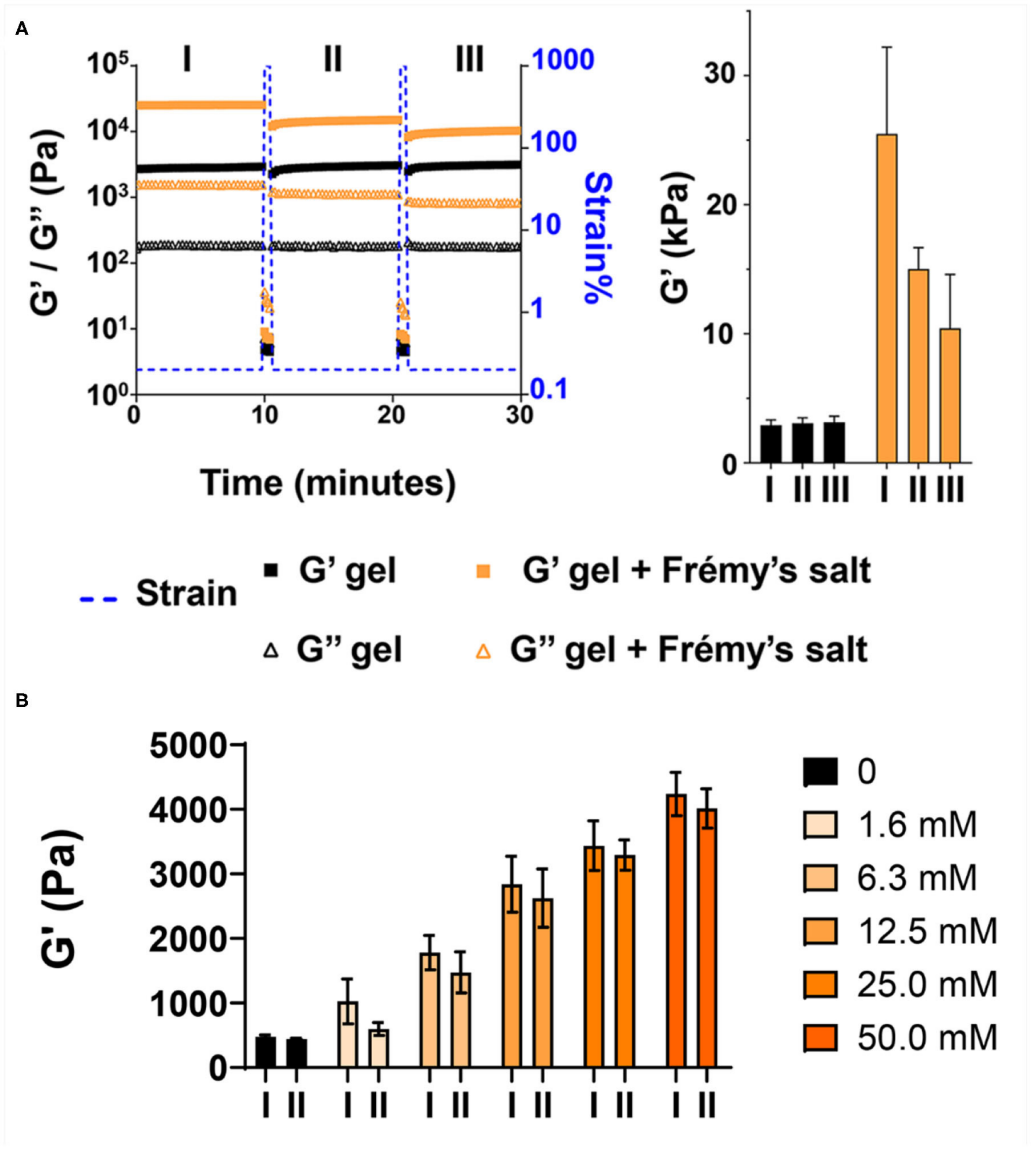
Frémy's salt increases the rigidity of tyrosine-functionalized gels. **(A)** Rheological characterization of 1 wt% gels after overnight incubation with or without Frémy's salt. Gels were subjected to repetitive shear-thinning cycles. In each cycle the storage (G′) and loss (G″) modulus of the gels were monitored as follows: 10 min of measurements within the linear viscoelastic regime (LVE, 0.2% strain, 6 rad/s), then 30 s under high strain that rupture the gel network (1,000% strain, 6 rad/s), following by 10 min measurements of the G′/G″ again within the LVE regime (0.2% strain, 6 rad/s) where the gel recovers. The bar graph at right displays G′ values obtained at the end of each 10 min measurement (marked as I, II, III). **(B)** Rheological characterization of 0.5 wt% gels after overnight incubation with different concentrations of Frémy's salt. Bar graph marked as I displays G′ values obtained at the end of the first 10 min measurement. Bar graph marked as II, displays recovery G′ values of the gels obtained 10 min after applying high strain (1,000%) to thin the gel network.

We then wanted to examine how tyrosine oxidation affected the local morphology of the fibrils and the mechanical properties of the gel matrix. First, TEM was used to study the underlying structural characteristics of the fibrils. Fibrils were isolated from gel via diluting the gel with water to allow visualization of distinct fibrils. As seen in Figure 3A and Supplementary Figure 5A, in the absence of Fremy's salt, the fibrils that constituted the gel network display well-defined uniform morphology of mostly discrete long fibrillar structures that are approximately 3 nm in width. In contrast, following an overnight oxidation with Frémy's salt, though the gel was water-diluted, most of the fibrils were no longer distinct from each other and rather appear as clusters of entangled fibrils (Figure 3B, Supplementary Figure 5B). The fibrils were of inconsistent length (Supplementary Figure 5B), mostly shorter than fibrils isolated from an unoxidized gel, but with a similar characteristic width of about 3 nm. Collectively, the TEM data suggests that the local morphology of fibrils defined by the arrangement of assembled peptides is not influenced by oxidation. Although we cannot rule out surface-effects by the grid, oxidation does seem to influence the interactions between fibrils, as evident by their clumping. Thus, introducing the Frémy's salt to the established gel does not compromised the already assembled fibril, rather most likely introduces inter- or intra-fibril covalent crosslinks via oxidized phenol groups. Preservation of ordered molecular nanostructures was also reported in other post-self-assembly crosslinking systems (Zhang et al., 2012; Wei et al., 2016). Since introducing such covalent crosslinks can increase the cohesion of the supramolecular network, we wanted to examine the effect of Frémy's salt on the mechanical rigidity of the gels. Rheological studies were performed on oxidized and unoxidized tyrosine gels (Figure 4). To ensure homogenous oxidation of tyrosine throughout the gel, 300 μL of concentrated Frémy's salt solution (100 mM; ~95 eq) was added to the top of the gels. To account for any possible contribution of an increase in ionic strength to gel rigidity, 300 μL of a 100 mM NaCl solution was added to the control gels containing no Frémy's salt. Prior to each measurement the salt solutions above the gels (either Frémy or NaCl) were removed, and the gels were washed. Measurements of storage and loss modulus (G′ and G″, respectively) of pre-formed gels, collected in the linear viscoelastic (LVE) regime (0.2% strain, 6 rad/s, Supplementary Figure 6) display a significant increase in gel rigidity once the gel is oxidized by Frémy's salt, as indicated by the differences in G′ between unoxidized and oxidized gel. Initial G′ values of oxidized gels were about eight times higher than G′ values of unoxidized gels (25,470 ± 6,723 vs. 2,932 ± 401 Pa, respectively). Repetitive shear-thinning cycles were also performed to study the material's ability to recover after thinning. Here, following examination of the gel G′/G″ within the LVE, high strain (1,000% strain) is applied to shear-thin the gel. The high strain is then decreased and the gel G′/G″ values are recorded again within the LVE regime. Upon performing several shear cycles (Figure 4A), we observe that both the oxidized and the unoxidized gels were capable of shear-thin/recovery behavior. While the unoxidized gel fully recovered to its pre-sheared G′ values (105 ± 3%), the oxidized gels did not, showing about 60% recovery in the first shearing cycle with greater deviation between the samples (61 ± 13%). This gradual decrease in G′ as a function of thinning cycles indicates that the crosslinked network, although more rigid than non-chemically crosslinked material, losses some of its mechanical integrity when shear-thinned. Yet, it is worth mentioning that the recorded recovered rigidity values of the oxidized gels were still higher than the unoxidized gels. Further, this rheological behavior is conducive to syringe-delivery of the crosslinked gel for applications that demand its local placement.

The enhancement of the storage modulus is dependent on the concentration of Frémy's salt used for crosslinking. Figure 4B shows that at low concentrations (1.6 and 6.3 mM), only a small enhancement is realized, which increases with increasing concentrations of the salt. As discussed above, a change in material color from red to orange-yellow indicates the installment of crosslinks. Thus, the spatial distribution of coloration within the gel provides insight into the distribution of crosslinks throughout the material. When an excess amount of Frémy's salt (molar ratio of 33:1; Frémy's salt:Tyr) is used, uniform coloration is observed suggestion that crosslinks have been installed homogenously throughout the gel (Figure 2A, Supplementary Figure 7). In contrast, when a molar ratio of ~1:1 or 4:1 is used, coloration is only observed near the surface of the gel, Figure 4B. This indicates that at low concentrations of oxidant there is not enough to permeate the entire gel volume and crosslinks are only installed proximal to the solution-gel interface, Supplementary Figure 7.

**Figure 5:**
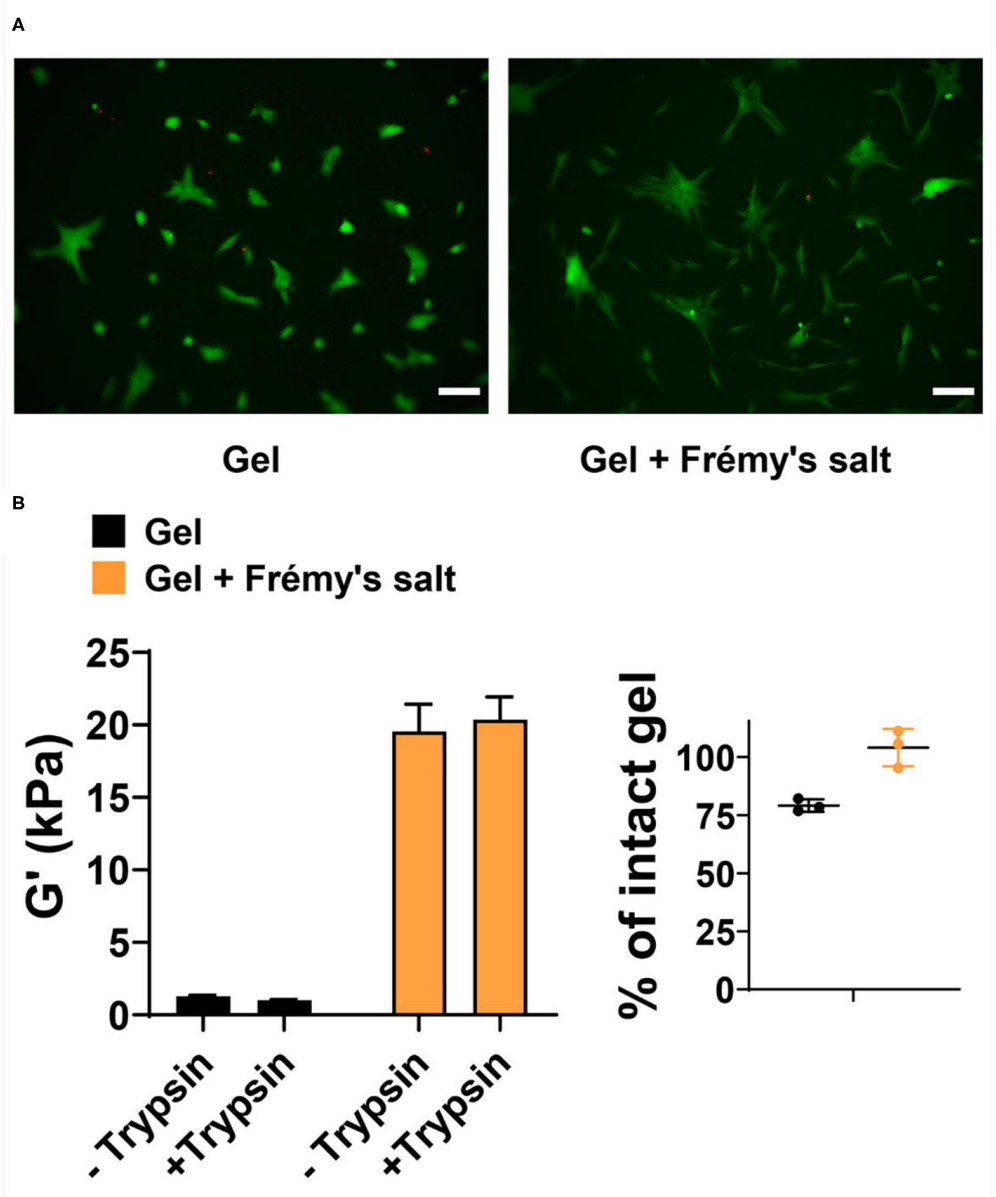
**(A)** Live/Dead assay of human dermal fibroblasts 48 h after being seeded onto 1 wt% tyrosine-gels untreated or treated with Frémy's salt (left and right panels, respectively). In live cells enzymatic conversion of non-fluorescent calcein-AM to calcein produce green fluorescence, whereas dead cells are labeled with ethidium homodimer-1 and fluoresce red. Scale bar is 100 μm. **(B)** Degradation of Frémy's salt-treated or untreated gels by trypsin. Rheological measurements were performed on 1 wt% gels following 2 days of incubation with trypsin or buffer as a control. Percentage of intact gel was calculated by dividing the G′ value of trypsin treated gel with the average of untreated control gels. Individual values of trypsin-treated gels are plotted with the mean and standard deviation.

Lastly, we evaluated the cytocompatibility of Frémy's salt-treated gels toward human dermal fibroblast (HDF). Frémy's salt is intended to increase the mechanical rigidity of tyrosine gels upon demand, *in vitro*, prior to the ultimate usage of the gels. For example, one can use Frémy's salt to increase the rigidity of gel matrix for 2D cell growth. Here, Frémy's salt can be added to the gel to increase its rigidity and simply removed before plating cells on the material. Figure 5A shows a Live/Dead cytocompatibility assay performed with Frémy's salt-treated or untreated tyrosine gels. In this assay, HDF cells are incubated on top of the gels and cell viability is assessed using fluorescence microscopy and fluorescent dyes, where live cells fluoresce green and dead cells fluoresce red. As seen in Figure 5A, cells are viable on both surfaces. Interestingly, cell morphology and spreading on each surface are slightly different. This might be attributed to changes in gel properties such as surface topography and/or mechanical rigidity that result from gel oxidation. We should note that Frémy's salt on its own is cytotoxic, so the gels must be washed before cells are introduced to the material. We also examined the enzyme-mediated degradation of Frémy's salt treated gels by measuring their storage modulus after 2 days of incubation with trypsin. Figure 5B demonstrates that both oxidized and unoxidized gels are relatively stable. However, the oxidized gel is more stable having not degraded significantly at day 2. Although not investigated here, longer times most likely would result in further degradation.

In conclusion, we present the design and characterization of a peptide-based gel system whose mechanical rigidity can be increased on demand, by chemically introducing covalent crosslinks within the supramolecular gel network. The addition of Frémy's salt to the gel enhanced its rheological properties, while conserving its shear-thinning behavior and local morphological fibrillar structure. Moreover, the Frémy's salt-treated gels are cytocompatible toward HDF cells and therefore may find future uses in biomedical applications such as scaffolds for cell growth. In general, this simple chemical approach can be used to modulate the mechanical rigidity of peptide-based self-assembled gels and complements the use of enzymes (e.g., tyrosinase) to oxidize tyrosine residues within gel systems. Future directions will include examination of Frémy's salt contribution to the mechanical rigidity of tyrosine gels as a function of tyrosine content and location within the peptide gelator.

## Materials and Methods

### Materials

Rink amide ChemMatrix® resin, Oxyma, and all other Fmoc-protected amino acids were purchased from Novabiochem®. 2-(6-Chloro-1H-benzotriazole1-yl)-1,1,3,3-tetramethylaminium hexafluorophosphate (HCTU) was purchased from Chem Impex International. Piperidine was purchased from Alfa Aesar. Ethanedithiol (98+%) was purchased from Fluka. Trifluoroacetic acid (TFA, 97%), anisole (99%), thioanisole (>=99%), 4-(2-hydroxyethyl)-1-piperazineethanesulfonic acid (HEPES), and potassium nitrosodisulfonate (Frémy's Salt, 220930) were purchased from Sigma Aldrich. N,N′-Diisopropylcarbodiimide (DIC, 99%), N,N-Diisopropylethylamine (DIEA), Dimethylformamide (DMF, 99.9%), Dichloromethane (DCM, >=99.8%), Diethyl ether and acetonitrile were purchased from Fisher Scientific.

### Peptide Synthesis

The tyrosine-containing peptide was synthesized by standard Fmoc-solid phase peptide synthesis using a Liberty Blue™ automated microwave peptide synthesizer (CEM) with H-Rink amide ChemMatrix® resin. Resin-bound peptide was cleaved and side chain-deprotected using a cleavage cocktail of TFA:thioanisole:ethanedithiol:anisole (90:5:3:2) for 3 h under argon. Crude peptide was purified by RP-HPLC using a preparative Vydac C18 peptide column at 40°C. Solvents for RP-HPLC consisted of standard A (0.1% TFA in water) and standard B (0.1% TFA in 9:1 acetonitrile/water). Gradients were used as follows: an isocratic gradient from 0 to 2 min at 0% standard B, a linear gradient from 0 to 20% standard B for 10 min and a linear gradient of 20 to 100% standard B over an additional 160 min. The peptide eluted at approximately 34% B, lyophilized and then analyzed using analytical HPLC and LC-MS. Analytical HPLC chromatograms and ESI (+) mass spectra of the pure peptide are provided in Supplementary Figure 8.

### Gel Preparation

Gels were prepared by dissolving lyophilized peptide in water to obtain 2X concentrated (wt%) peptide stock solution. Peptide assembly was initiated by mixing together equal volumes of peptide stock solution and chilled 2X HEPES buffer solution (150 mM HEPES, 300 mM NaCl, pH 7.4) on ice. The final mixed solution (1X wt% peptide in 75 mM HEPES, 150 mM NaCl, pH 7.4) was then incubated for 2 h at 37°C, resulting with a self-supporting gel.

### Gel Oxidation by Frémy's Salt

Tyrosine gels were prepared as described, where following 2 h incubation at 37°C, Frémy's salt in HEPES buffer (75 mM HEPES, 150 mM NaCl, pH 7.4) was added on top of the gel and incubated overnight at 37°C. Typically, the Frémy's salt solution was added at molar concentration that is about 32 times higher than the final molar concentration of the peptide and in a volume that is 3 times higher than the final gel volume [e.g., on top of 100 μL of 1 wt% gel (~3.15 mM), 300 μL of 100 mM Frémy's salt in HEPES buffer were added].

### Monitoring Color Change in Oxidized Gels

To monitor color change in oxidized gels using the naked eye, 1 wt% tyrosine-gel in HEPES buffer was prepared as described above where 10 μL aliquots were transferred to Eppendorf tubes for 2 h incubation at 37°C. Thirty microliter of either 100 mM Frémy's salt or 100 mM NaCl, as a control, dissolved in HEPES buffer (75 mM HEPES, 150 mM NaCl, pH 7.4) were added on top of the gels and pictures of the tubes were taken at different time points. To monitor color change using UV-Vis 0.125 wt% tyrosine-peptide gel in HEPES were prepared as described above in a 96-well plate (each well had a final volume 80 μL). Following 2 h incubation at 37°C, 8.33 mM Frémy's salt in HEPES buffer was added to each well and absorption spectra were collected from 270 to 700 nm using a plate reader (Epoch™ Microplate Spectrophotometer, Biotek).

### Transmission Electron Microscopy

1 wt% gels were prepared as described above in Eppendorf tubes, incubated overnight at 37°C with or without 100 mM Frémy's salt. The samples were prepared by diluting the gels X50 into water to allow visualization of distinct fibers. A 5 μL drop of peptide solution was placed on a 200-mesh copper grid covered by carbon film (Electron Microscopy Science) for 1 min, then blotted by filter paper. Subsequently, 5 μL of 0.75% uranyl formate was added to the grid and allowed to stand for 1–2 min, then blotted with a filter paper and left to air dry. Images were taken with a Hitachi 7650 at 80 kv accelerating voltage. Average fibril width was measured via ImageJ software by taking 164 and 156 independent measurements of distinct fibrils of the non-oxidized and oxidized gel, respectively.

### Rheology

Rheological measurements were performed on pre-formed gels using an AR G2 rheometer (TA Instruments) equipped with an 8-mm stainless steel parallel plate geometry tool. Hundred microliter of 1 wt% tyrosine-gels in HEPES buffer were prepared as described above in Corning® Costar® Transwell® cell culture inserts positioned in a 24-well plate. Following the 2 h incubation at 37°C 300 μL of either 100 mM Frémy's salt or 100 mM NaCl solutions (75 mM HEPES buffer containing 150 mM NaCl, pH 7.4) were added on top of the gels for overnight incubation (~16 h) at 37°C. The next day, the solution on top of the gel was removed, the gels were washed, and the rheological properties of the gels were determined. Shear-thinning/recovery cycles were performed as follows: 10 min time-weep at the LVE regime (0.2% strain, 6 rad/s), then shearing for 30 s under high strain (1,000% strain, 6 rad/s), following by a 10 min time-sweep at the LVE regime (0.2% strain, 6 rad/s). A dynamic frequency sweep in the frequency range of 0.1–100 rad/s was collected at 0.2% strain and a dynamic strain sweep (0.1–1,000 strain%) was collected at a constant frequency of 6 rad/s. Rheological data represent the average G′ and G″ obtained from at least three independent measurements. Frémy's salt concentration dependent studies were done similarly with pre-formed 0.5 wt% tyrosine gels and different concentrations of Frémy's salt dissolved in HEPES buffer. The control tyrosine-gel (0 mM Frémy's salt) was supplemented with additional 50 mM NaCl.

### Cell Viability Assay

1 wt% gels were prepared as described above in a 96-well plate (50 μL per well). Following 2 h of incubation at 37°C, 200 μL of HEPES buffer (75 mM HEPES, 150 mM NaCl, pH 7.4) either with or without 100 mM Frémy's salt were added on top of the gels for overnight incubation (~16 h) at 37°C. Buffer solution from the top of the gels was removed and gels were washed several times with fresh HEPES buffer (25 mM HEPES, 150 mM NaCl, pH 7.4). Human dermal fibroblast (HDF, ATCC® PCS-201-010™) cells were trypsinized and counted using Bio-Rad TC20™ Automated Cell Counter. The resulting cell suspension was diluted with serum containing Dulbecco's Modified Eagle's Medium (DMEM, Gibco™ GlutaMAX™, 10567-014 supplemented with 50 mg/mL gentamicin). Two hundred microliter of 10,000 HDF cells were placed on top of each gel and on a control tissue-culture treated polystyrene surface. After 48 h of incubation at 37°C and 5% CO_2_, the medium was removed, and each well was washed with serum-free DMEM medium. Cell viability was evaluated using LIVE/DEAD™ Viability/Cytotoxicity assay (Molecular Probes, L3224) according to manufacture instructions. Typically, 100 μL serum-free DMEM medium containing both 1 μM calcein AM and 2 μM ethidium homodimer was added into each well and incubated at 37°C for 15 min. Cells were imaged using fluorescence microscopy (EVOS FL Cell Imaging System, Thermo Fisher Scientific). Samples were prepared in quadruplicate with two biological repeats. Images of merged green and red channels were prepared using ImageJ software. Brightness and contrast were adjusted for the figures as follows: minimum and maximum displayed values of 10 and 65 for the red channel. Minimum and maximum displayed values of 10 and 130 for the green channel.

### Proteolytic Degradation of Gels

Eighty microliter of 1 wt% tyrosine-gels in HEPES buffer were prepared as described above in Corning® Costar® Transwell® cell culture inserts positioned in a 24-well plate. Following the 2 h incubation at 37°C, 300 μL of HEPES buffer (75 mM HEPES, 150 mM NaCl, pH 7.4) with or without 100 mM Frémy's salt were added on top of the gels for overnight incubation (~16 h) at 37°C. The next day, buffer solution was removed from the gels and all gels were washed with HEPES buffer supplemented with EDTA (25 mM HEPES, 150 mM NaCl, 1 mM EDTA pH 7.8). Bovine pancreatic trypsin (Sigma, T9201) was prepared in buffer (50 mM BTP 150 mM NaCl 1 mM EDTA pH 7.8) and introduced to the top of the gels yielding a final trypsin concentration of 125 nM upon equilibrium. Gels were incubated for 2 days at 37°C. Control gels (oxidized and non-oxidized) were prepared in the same manner in the absence of trypsin. Following 2 days, solution from the gel was removed and rheological measurements were performed.

## Data Availability Statement

The original contributions presented in the study are included in the article/Supplementary Materials, further inquiries can be directed to the corresponding author.

## Author Contributions

GF and JS designed the experiments and prepared the manuscript. GF performed the experiments. All authors contributed to the article and approved the submitted version.

## Conflict of Interest

The authors declare that the research was conducted in the absence of any commercial or financial relationships that could be construed as a potential conflict of interest.
