# Supplementary material for: Utilizing Frémy's Salt to Increase the Mechanical Rigidity of Supramolecular Peptide-Based Gel Networks
Source: Front Bioeng Biotechnol. 2021 Jan 5;8:594258. doi: 10.3389/fbioe.2020.594258 (PMC7813677; doi:10.3389/fbioe.2020.594258)
Supplement: Supplementary file 1 [file Data_Sheet_1.pdf]

supplementary

Utilizing Frémy's Salt to Increase the Mechanical Rigidity of Supramolecular Peptide-Based Gel Networks

Galit Fichman[1] and Joel P. Schneider[1*]

[1]Chemical Biology Laboratory, National Cancer Institute, National Institutes of Health, Frederick, Maryland, USA

*** Correspondence:** Schneiderjp@mail.nih.gov

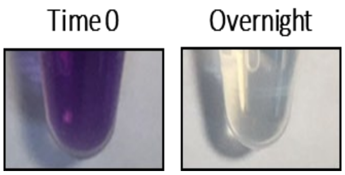

Figure S1. Color change of Frémy's salt solution. As Frémy's salt dissolved in HEPES buffer (75 mM HEPES, 150 mM NaCl, pH 7.4) the solution turns purple due to the presence of nitrosodisulfonate ions. Overtime the nitrosodisulfonate ions decompose and the purple color of the Frémy's salt solution is discharged.

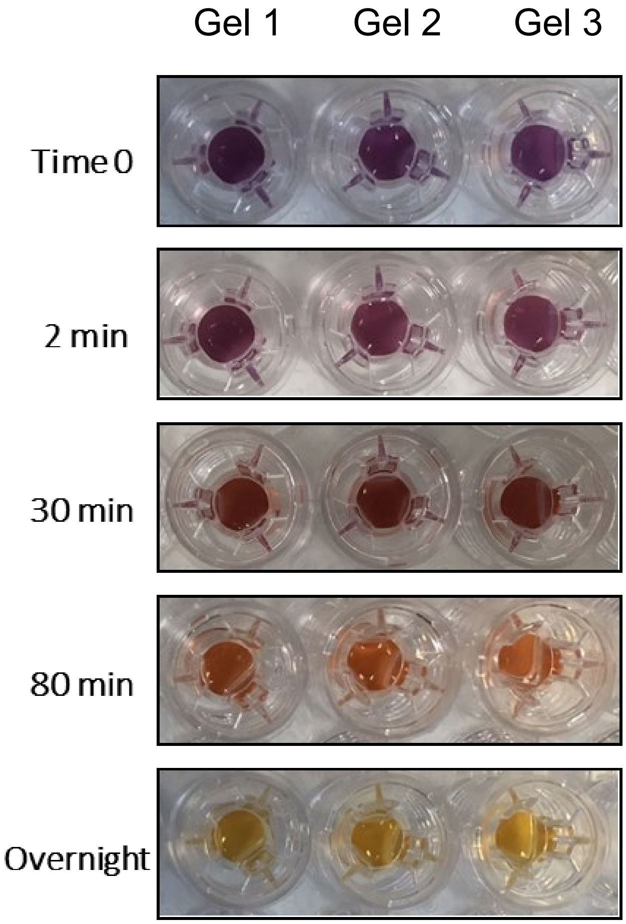

Figure S2. Top view of the color change that is observed overtime when 100 mM Frémy's salt solution is added to 1wt % (~ 3mM) tyrosine-functionalized gels (both gels and the Frémy's salt solution are in HEPES buffer (75 mM HEPES, 150 mM NaCl, pH 7.4)). Change in color for both the gel and the solution on top of each gel was followed as a function of time.

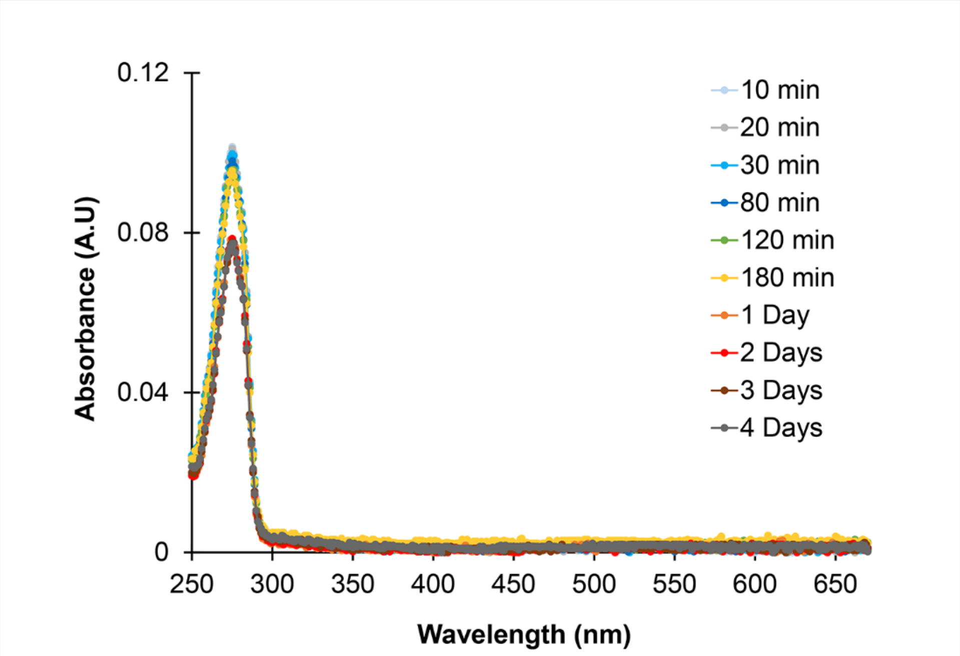

Figure S3. UV-vis spectra of 0.125wt% tyrosine-functiolized peptide in HEPES buffer (75 mM HEPES, 150 mM NaCl, pH 7.4) collected at different time points.

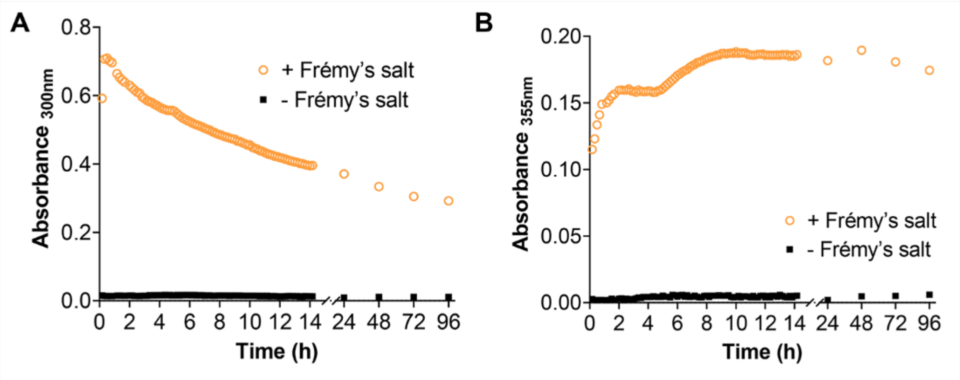

Figure S4. Change in absorbance of 0.125wt% tyrosine-functionalized peptide in HEPES buffer (75 mM HEPES, 150 mM NaCl, pH 7.4) as a function of time, in the presence or absence of Frémy's salt, at **A**. 300 and **B.** 355nm

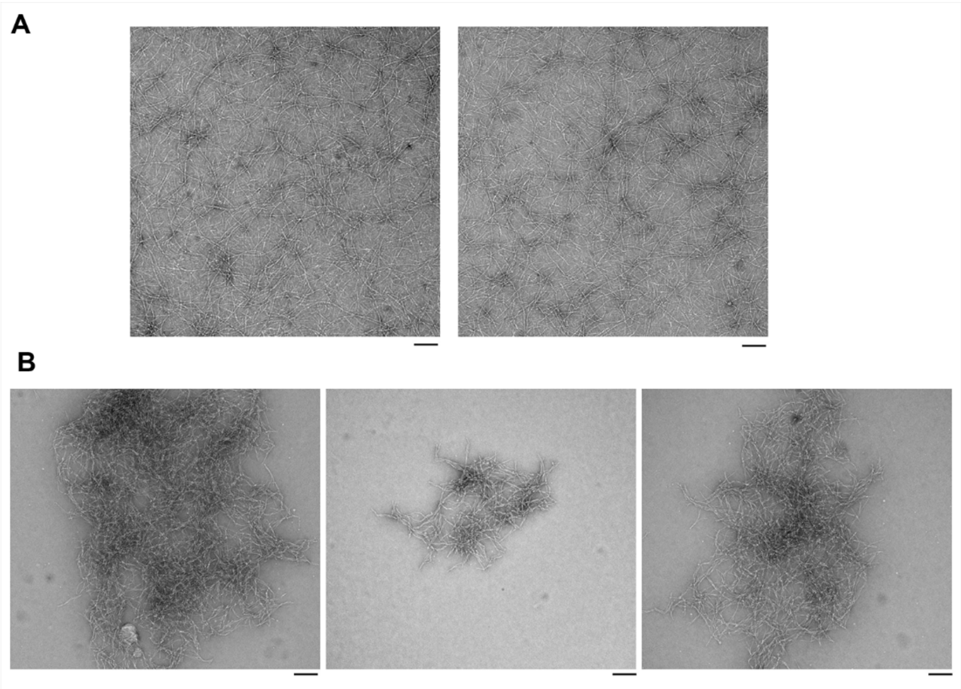

Figure S5. TEM micrographs showing fibrils isolated from 1 wt% fibrillar gel networks (**A**) in the absence of oxidant or (**B**) following overnight oxidation with Frémy's salt. Scale bar =100 nm.

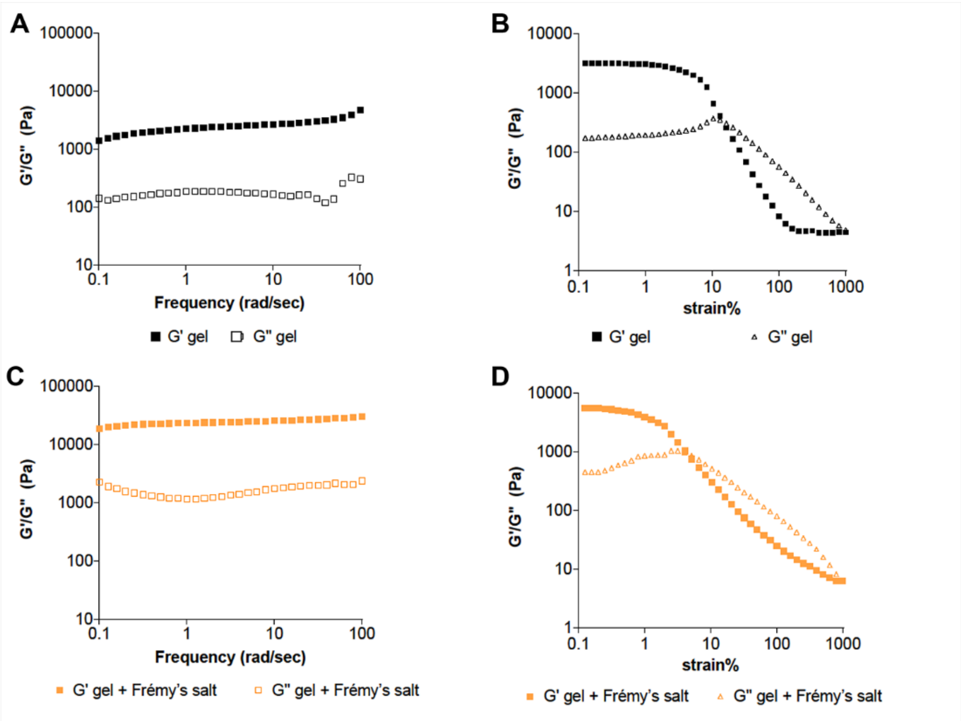

Figure S6. Frequency and strain sweep of pre-formed 1wt% peptide gel in HEPES buffer, following an overnight incubation without (**A, B**) or with Frémy's salt (**C, D**). Frequency sweep data was collected at 0.2% strain, strain sweep data was collected at a frequency of 6 rad/sec.

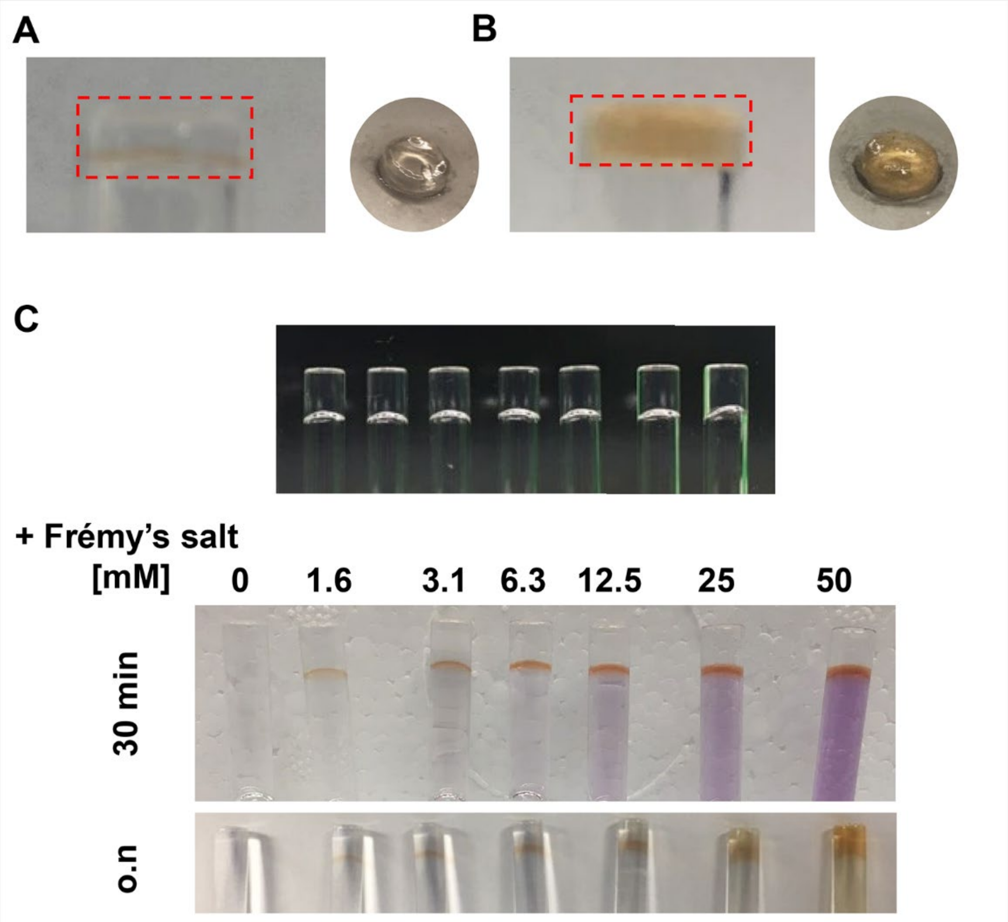

Figure S7. Images of pre-formed 0.5wt% gels following overnight incubation in the presence of (**A**) 1.6 or (**B**) 50 mM Frémy's salt. In the presence of 1.6 mM coloration is only observed near the solution-gel interface. (**C**) Monitoring concentration-dependent permeation of Frémy's salt into pre-formed gels in glass vials. 300 μL of HEPES buffer containing different concentration of Frémy's salt were added on top of 100 μL pre-formed 0.5wt% gels. Following the addition of Frémy's salt, gels were incubated at 37 °C and images of the vials were taken after 30 minutes and ~16 hours of incubation.

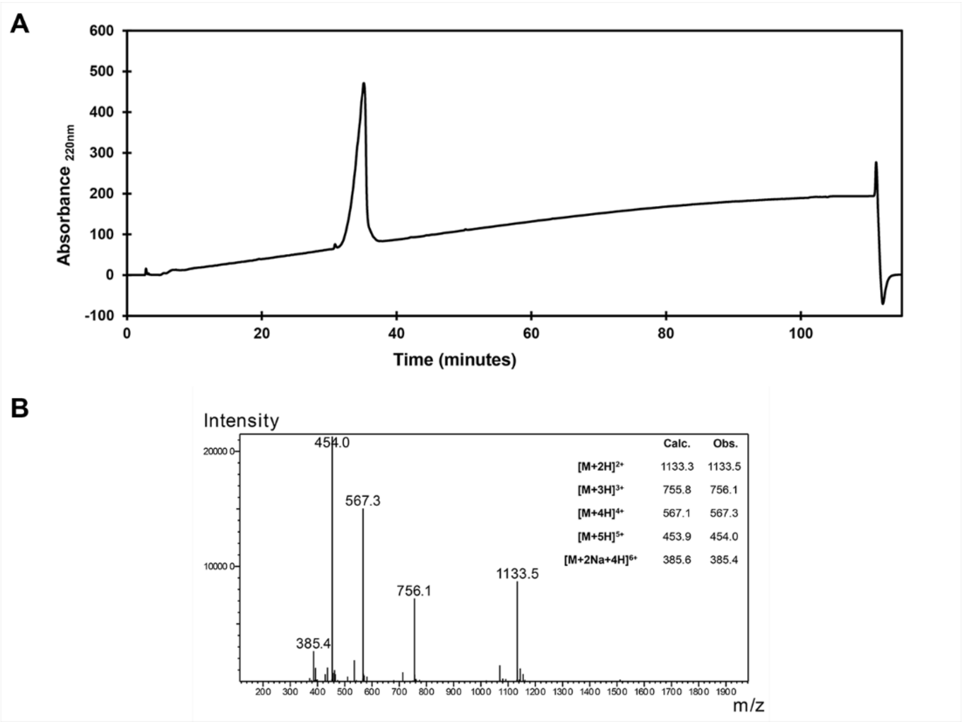

Figure S8. (**A**) Analytical HPLC (0%-100% B over 100 min) and (**B**) ESI (+) mass spectrum of purified tyrosine-gel.
